# Synchronized acoustic emission and high-speed imaging of cavitation-induced atomization: The role of shock waves

**DOI:** 10.1016/j.ultsonch.2025.107233

**Published:** 2025-01-18

**Authors:** Abhinav Priyadarshi, Paul Prentice, Dmitry Eskin, Peter D. Lee, Iakovos Tzanakis

**Affiliations:** a School of Engineering Computing and Mathematics, Oxford Brookes University, Oxford, UK; b Cavitation Laboratory, Centre for Medical and Industrial Ultrasonics, University of Glasgow, Glasgow, UK; c Brunel Centre for Advance Solidification Technology (BCAST), Brunel University of London, Uxbridge, UK; d Department of Mechanical Engineering, University College London, London, UK; e Research Complex at Harwell, Harwell Campus, Oxfordshire, UK; f Department of Materials, University of Oxford, Oxford, UK

**Keywords:** Ultrasonic atomization, Cavitation bubbles, water, Isopropyl alcohol, Glycerol, high-speed imaging, Acoustic emissions, Shock waves

## Abstract

This study experimentally investigates the role of cavitation-induced shock waves in initiating and destabilizing capillary (surface) waves on a droplet surface, preceding atomization. Acoustic emissions and interfacial wave dynamics were simultaneously monitored in droplets of different liquids (water, isopropyl alcohol and glycerol), using a calibrated fiber-optic hydrophone and high-speed imaging. Spectral analysis of the hydrophone data revealed distinct subharmonic frequency peaks in the acoustic spectrum correlated with the wavelength of capillary waves, which were optically captured during the onset of atomization from the repetitive imploding bubbles. This finding provides the first direct evidence that the wavelength of the growing surface waves, which governs capillary instability resulting in droplet breakup, is linked to the periodicity of shock waves responsible for the onset of the subharmonic frequencies detected in the acoustic spectra. This work contributes to a deeper understanding of ultrasonic atomization, signifying the role of cavitation and shock waves in the atomization process.

## Graphical abstract

**Figure:**
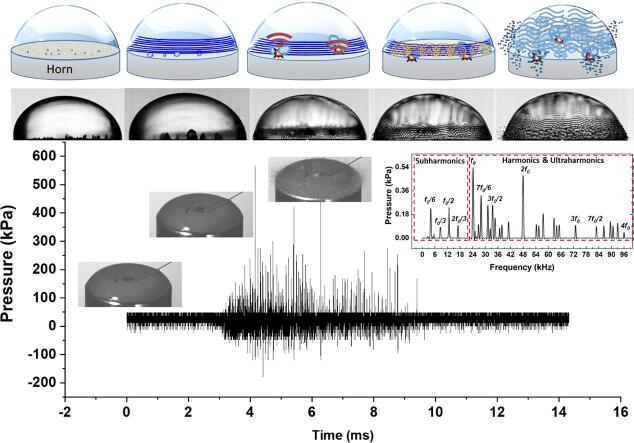
Role of shock waves in triggering atomization deciphered through opto-acoustic characterization.

## 1 Introduction

Ultrasonic atomization is a widely adopted technique for generating fine sprays, powder feedstock and aerosols of liquids, with applications spanning various industries, including food, pharmaceuticals, combustion, coatings, and, more recently additive manufacturing [1], [2], [3], [4]. Ultrasonic atomization uses high-frequency acoustic waves in the range of 20 kHz and above to excite capillary waves on the liquid surface, leading to detachment of liquid droplets from the wave crests. The characteristics of these capillary waves, such as their frequency and wavelength, are influenced by various factors, including the intensity of the ultrasonic vibrations, the properties of the liquid (e.g., viscosity, surface tension), and the geometry of the liquid surface [5], [6], [7], [8]. Recent studies [9], [10] have suggested that the inertial cavitation is a key driver of capillary wave instability, directly or indirectly supporting the conjunction theory, where the intense hydraulic shock waves emitted upon bubble implosion excite and amplify the capillary waves, eventually driving atomization as previously proposed by Boguslavskii and Eknadiosyants [11]. Several studies have also analytically modelled the surface wave instability on a liquid surface subjected to vertical vibrations revealing that the frequency of the most unstable interfacial wave mode is subharmonic to the driving acoustic frequency [8], [12], [13], [14]. Recently, Panda et al. [15], studied the dynamics of vibrating sessile drops and found that the subharmonic azimuthal (non-axisymmetric) waves superimpose on the harmonic axisymmetric waves leading to chaotic mixing that drive the formation of droplets. However, there is hardly any explanation offered for the formation of these subharmonic modes that eventually promote atomization. Despite the well-established role of capillary waves in inducing atomization, direct experimental evidence causally linking cavitation dynamics and the corresponding shock wave emissions to the formation and growth of surface waves (at subharmonic wavelengths) remains elusive in the current body of literature. Some theoretical studies have already indicated that shock waves from periodically expanding and collapsing bubbles lead to the generation of surface disturbances on the liquid–air interface in the form of capillary waves [16], [17], [18]. To date, only the study by Galleguillos [19] involved experimental acoustic detection during ultrasonic atomization. However, no signs of cavitation were observed in this work. On the other hand, in our recent study [9] we used synchrotron X-ray visualization to show that cavitation is indeed present and precedes the formation of capillary waves; however, a direct link was not established.

In this paper, we aim to quantify the relationship between bubble collapse events and cavitation-induced shock wave emissions along with the spatio-temporal evolution of surface waves preceding ultrasonic atomization of deionized water, isopropyl alcohol, and glycerol. The selection of these liquids for this study was motivated by their distinct physical properties and relevance to understanding of ultrasonic atomization mechanisms. The liquids represent a range of viscosities from low (water) to intermediate (isopropyl alcohol) and high (glycerol), facilitating the investigation of the influence of viscosity on cavitation, shock waves, and atomization. These liquids also exhibit different surface tensions and vapor pressures, which affect cavitation activity and atomization behaviour as previously examined by Eisenmenger [20]. Moreover, these liquids are used in diverse applications like solvents, cleaners, and pharmaceuticals, making the findings broadly relevant. Thus, by synchronizing high-speed visualization with calibrated acoustic monitoring, we can characterize the relationship between the frequency of the evolving capillary waves (observed optically) and the registered shock waves (detected acoustically). The frequency of these shock waves is represented by the appearance of the subharmonic signatures (*f*_0_/*m*, where *m* is integer) in the acoustic noise spectrum [21], [22], which are closely linked to the periodic collapses of cavitation bubble clouds. Our findings also provide direct validation of the cavitation – capillary wave combination hypothesis that could be valuable in optimizing the ultrasonic atomization process for various applications, enhancing control over droplet size distribution, spray patterns, and overall efficiency.

## 2 Experimental setup

**Fig. 1:**
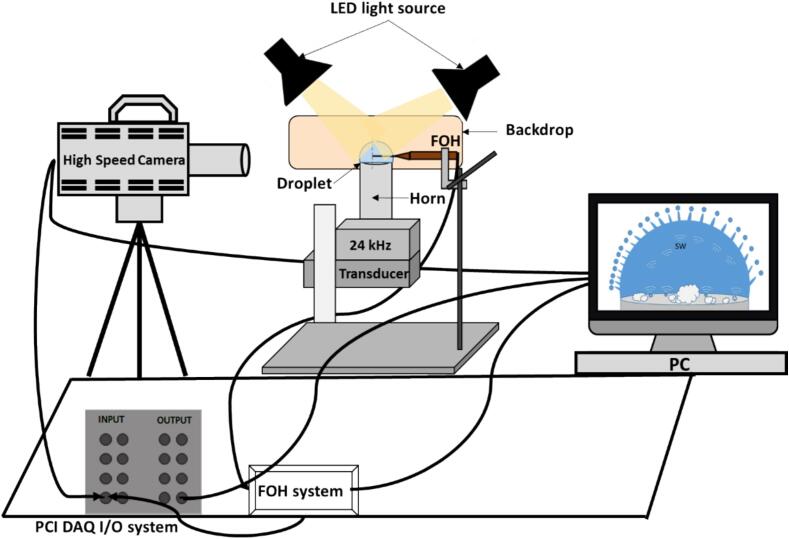
Schematic representation of the experimental test rig used for imaging and acoustic monitoring of ultrasonic atomization of a sessile droplet.

The experiments utilized a custom test rig with a piezoelectric transducer operating at 24 kHz and an attached Ti horn tip of 7 mm diameter in an upright position (Fig. 1). Small amounts of deionized water (DIW), isopropyl alcohol (IPA), and glycerol (Gly) were placed onto the horn tip surface to create droplets or films with varied thickness and/or contact angles. These were categorized as low (LA ≤ 20°), medium (MA ∼ 30–60°), and high (HA ∼ 60–90°) contact angle. This allowed us to assess interfacial confinement effects on cavitation activity and wave dynamics. To control the contact angle, we manually adjusted the droplet profile by precisely depositing specific volumes of liquid onto the horn tip, allowing surface tension forces to naturally establish the desired contact angle. We then fine-tuned the contact angle using a micro-pipette to ensure consistency and reproducibility throughout the experiment. The distinct physical properties of studied liquids at room temperature can be found in Table 1. Sessile droplets were ultrasonically excited at 20 % of the transducer rated power, corresponding to a peak-to-peak vibration amplitude of 25 μm. High-speed visualization of droplet/film surface dynamics was conducted using a Photron FASTCAM SA-Z camera at 100,000 frames per second (fps) and a resolution of 384 × 360 pixel, facilitated by a Navitar 12 × zoom lens and diffuse LED illumination.

**Table 1:** Physical properties of the liquids used in this study [24], [25], [28].

**Liquids**	**Density (kg/m^3^)**	**Viscosity (mPa.s)**	**Surface tension (N/m)**	**Vapour pressure (Pa)**
**Deionized water**	1000	1	0.072	2.2 × 10^−3^
**Isopropyl alcohol**	786	2.4	0.022	5.3 × 10^3^
**Glycerol**	1260	950	0.064	Negligible

Acoustic emissions from cavitation events within the droplet/film were simultaneously monitored using a calibrated fiber-optic hydrophone (FOH) (Precision Acoustics Ltd.) in direct contact with the liquid near the centre of the horn. The hydrophone, with a calibrated bandwidth of up to 30 MHz [23], was utilized to detect low frequency components and broadband shock waves emitted from inertial cavitation events [23], [24]. Acoustic data was acquired using a 14-bit PCI data acquisition system with a sampling rate of 20 MHz, synchronized with the high-speed camera. Raw acoustic signals obtained in voltage–time were processed using Fast Fourier Transformation (FFT) to obtain pressure (*P*) – frequency (*f*) spectra after taking into account the sensitivity of the hydrophone (see Fig. S1 in the supplementary material), revealing the cavitation activity signatures. Inverse FFTs were then applied to these acoustic spectra to reveal pressure (*P*) – time (*t*) domain signals as discussed in our earlier papers [23], [24], [25], [26], [27]. Acoustic detection was performed at least three times for each droplet case to ensure repeatability, consistent with high-speed imaging observations. Simultaneously, shadowgraphic high-speed image sequences were analyzed using a PFV4 video processing tool to extract the spatio-temporal evolution of capillary waves and their frequency/wavelengths on the droplet/film surface just before atomization is established.

## 3 Results and discussion

### 3.1 Capillary wave evolution

**Fig. 2:**
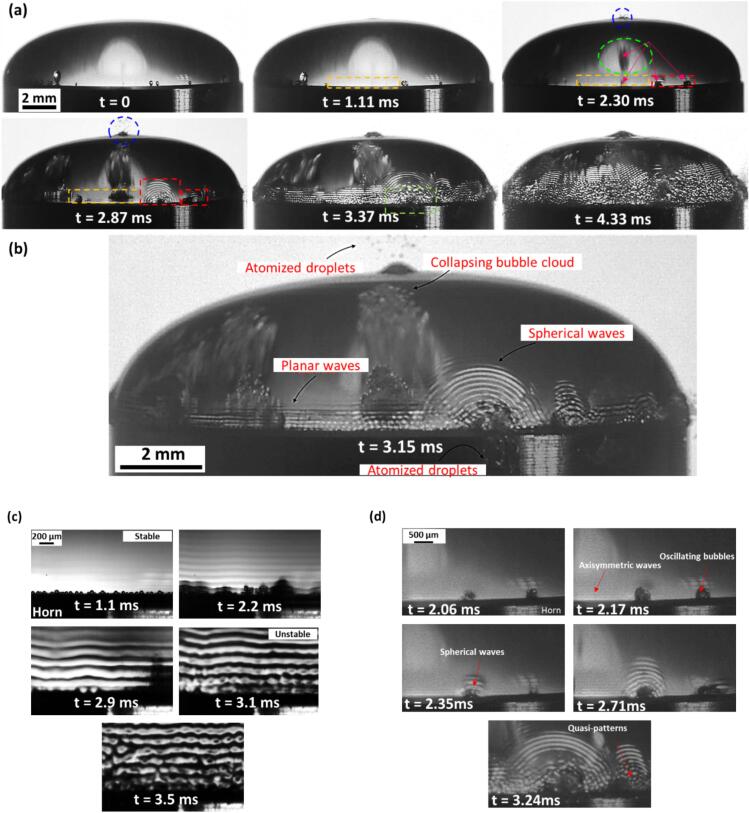
High-speed image sequence showing (a) initial stages of atomization, (b) a time instant showing formation of planar and spherical waves with collapsing bubble clouds and atomized droplets, (c) zoomed-in view of capillary wave evolution, and (d) interaction of planar (axisymmetric) waves with the spherical waves and their superimposition leading to surface wave instability, in a water droplet ultrasonically excited on a 14 mm horn tip.

The high-speed image sequence shown in Fig. 2a (refer to Supplementary Video 1), captured the initiation of atomization from a semi-spherical water droplet on an ultrasonic horn tip, exhibiting several critical stages in these first-time observations. At *t* = 0 ms, the droplet, containing multiple pre-existing bubbles, was at rest. Upon activating the ultrasound, these bubbles started to oscillate, split and coalesce, and faint planar (axisymmetric) stable surface ripples (enclosed in dashed yellow rectangle) emerged near the liquid-horn interface (*t* = 1.11 ms) likely due to the acoustic wave propagation from the source. As oscillations intensified (*t* = 2.30 ms), two notable phenomena were observed: (i) the formation of non-axisymmetric spherical (azimuthal like) surface wave patterns indicative of omnidirectional shock waves (enclosed in red dashed rectangle) generated by repetitive bubble collapses (indicated by arrows) in the vicinity of the liquid–air interface, and (ii) the rupture of the liquid–air interface at the top of liquid dome (encircled in blue), ejecting atomized droplets at speeds of 3–5 m/s even before the capillary waves were fully established. Note that the white central region (encircled in green) in Fig. 2a at *t* = 2.3 ms is the reflection from the high-power diffused LED light source used for illumination, while the dark region within this region is an oscillating bubble cloud (indicated by red arrows) located somewhere close to the midsection of the ultrasonic horn. Garen et al. [29], Zhang et al. [30], and Bempedelis et al. [31] observed similar phenomena using laser induced bubbles close to the liquid–air boundary, where the emitted shock waves were found to break the boundary to produce liquid jets. The rupture from the top of liquid dome is because of the strong bubble cloud implosions occurring axisymmetrically and in close proximity of the top region of the liquid–air interface as can be seen in Fig. 2b. This early rupture clearly shows the substantial role of cavitation-induced shock waves with possible contribution from high-speed microjets that can further interact with interphase boundary resulting in atomization. Subsequently at *t* = 2.87 ms, the oscillations became more vigorous, leading to further propagation of spherical waves and ejection of droplets from both the top of the dome and the bottom edge interface. By *t* = 3.37 ms, surface waves became more prominent, intense and destabilized following the interference between spherical and planar waves near the oscillating bubble cluster facilitating the formation of atomized jets (enclosed in green rectangle). The process continued and at *t* = 4.33 ms, intensified wave interactions and ongoing droplet breakup occur, particularly near the horn tip, showing the efficiency of ultrasonic energy in converting liquid mass into fine droplets. It is important to note here that droplet ejection initiated predominantly in regions where the spherical waves appeared upon bubbles collapsing in the vicinity of the liquid-horn-air interface as seen in Fig. 2b, thus providing a triggering mechanism for atomization, which was then amplified by the oscillating horn. This further reinforces our previous observations that cavitation is responsible for facilitating the atomization process. Another interesting observation was that the oscillation rate of the microbubbles, which was initially equal to the driving frequency of 24 kHz dropped to subharmonic frequencies close to 12 kHz (see Supplementary video 2) and was followed by destabilization of the surface ripples. Fig. 2c (refer to Supplementary Video 3) shows an image sequence of the zoomed-in view of the capillary waves transitioning from stable (*t* ≈ 1.1 ms) to unstable regime (*t* ≈ 3.1 ms). It is important to note here that this image sequence is a separate observation zooming in on the liquid–air interface, not a zoomed-in view of Fig. 2a, and captures the overall transition of surface waves from stable to unstable regimes prior to atomization. It is also interesting to note that the wavelength of the capillary wave increased with the number of acoustic cycles (horn tip oscillations), from approx. 105 µm to almost 208 µm (doubled) resulting in a departure from the fundamental frequency to the first subharmonic.

From these initial observations, we hypothesize that the main mechanism driving the formation of unstable surface waves and subsequent tiny droplets is the interaction of omnidirectional shock waves emitted from subharmonic bubble collapses at increased horn oscillations/cycles with stable axisymmetric (planar) waves propagating at liquid–air interface near the fundamental frequency. It should be emphasized here that every time a bubble or bubbly cloud collapses as seen in Fig. 2b, multiple bands of shock wave fronts are emitted. These shock waves, although had been resolved in our previous work [22], were difficult to observe through optical imaging in the present study because of the experimental limitations resulting from optical distortions and laser beam scattering. It is also worth noting that bubbles may also implode far from the boundaries of the liquid dome, such as in the middle of the sonotrode and since they are located farther from the liquid–air interface, their wave signatures (spherical wave patterns) do not appear on the liquid surface. The shock waves pulses from these distant collapses decay rather instantly within the first few hundred micrometers as observed elsewhere [32], contrasting with the bubbles collapsing in the vicinity of the interphase boundary. Thus, the circular wave patterns visible on the droplet surface can be interpreted as a blueprint of the shock waves periodicity emitted during each implosion inside the image frame. Interestingly, the wavelength of these spherical waves was approx. 148 µm, which coincided with the subharmonic frequencies of *f*_0_/2 in the acoustic spectrum (Section 3.3), being an acoustic signature of shock waves [22]. Meanwhile, the planar axisymmetric capillary waves travelling upwards from the horn surface had a wavelength close to 85 µm, aligning with the fundamental frequency of 24 kHz. As soon as the two wave types (planar/axisymmetric and spherical) interfered, destabilisation initiated as seen in Fig. 2d (see Supplementary Video 4). This interference triggered a chaotic wave pattern across the liquid dome which then ruptured to produce atomized droplets.

**Fig. 3:**
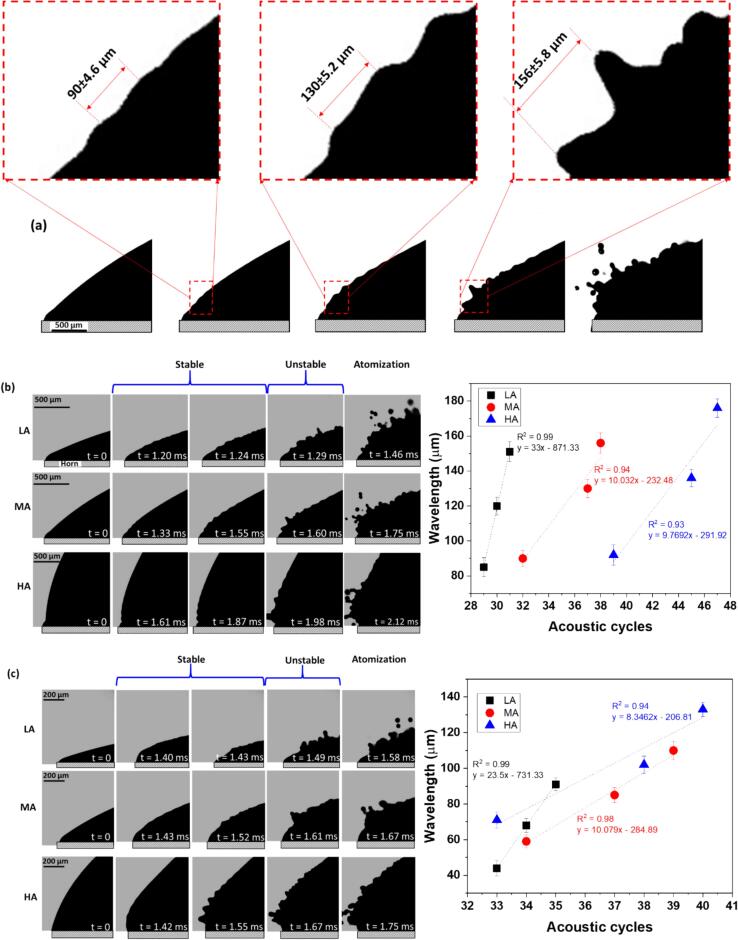

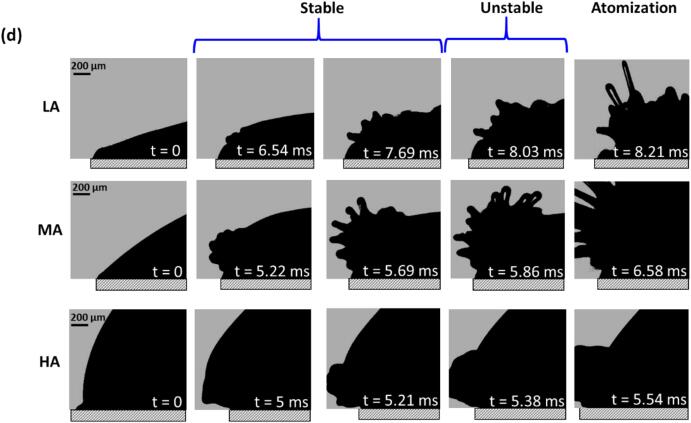
High-speed image sequence capturing the temporal evolution of capillary waves and atomization in liquid droplets. (a) representing the wavelength extraction method from the observed ripples on the interphase boundary, determined by visually measuring the distances between two consecutive crests, while (b), (c) and (d) correspond to DIW, IPA, and Gly, respectively, for different contact angles of the liquid droplet.

Let us now specifically look at the liquid–air interface of the droplets by imaging the spatio-temporal evolution of capillary waves formed close to the horn tip, for different thickness/contact angles in order to estimate their propagation frequency, until the point of wave instability prior to atomization as these were the regions that started to atomize first. The evolution of capillary waves was visualized for different liquids, i.e., DIW and IPA and Gly droplets as shown in Fig. 3. For each liquid, the wavelength of the observed ripples on the interphase boundary was determined by visually measuring the distances between two consecutive crests (see Fig. 3a) and was plotted with respect to the number of acoustic cycles providing a quantitative measure of wave evolution. To ensure accurate wavelength measurements, we selected frame by frame the crests that exhibited sharp and well-defined focal lines, and then averaged measurements from multiple crests for at least three similar observations. The crests that were chosen from the droplet surface were the ones that were located near the horn tip as this location exhibited the initial development and breakup of surface wave patterns upon ultrasonic excitation (see Supplementary Video 5). In addition to this, we also considered the significance of the cavitation regime development within the droplet and its role in the atomization process.

#### 3.1.1 Water

Fig. 3b depicts a high-speed photographic sequence of a DIW droplet subjected to ultrasonic excitation for different contact angles at 20 % of the input power. Initially, the droplet was in a state of rest, at *t* = 0. As ultrasonic vibrations were applied, the low angle (LA) droplet at *t* = 1.20 ms began to oscillate, marked by the first appearance of capillary waves with a wavelength, *λ_CW_* ≈ 85 µm corresponding to the driving frequency, *f*_0_. The phase velocity (*V*_CW_) of capillary wave propagation was experimentally measured to be approx. 2 m/s. These waves grew in wavelengths from 85 ± 5 µm to 120 ± 5 µm at *t* = 1.24 ms, indicating a shift in their propagation frequency to the subharmonic mode at 2*f*_0_/3, possibly resulting from the oscillation and collapse of cavitation clouds emitting shock waves at *f*_0_/3 as seen in [22]. Note, at each acoustic cycle a new ripple of specific wavelength evolved from the horn surface and propagated from the bottom to the top of the liquid dome, as discussed previously [33], [34]. The change in the wavelength of these ripples was most likely affected by a reduction in the oscillation and collapse frequency of the cavitation bubbles within the liquid, emitting omnidirectional subharmonic shock waves as discussed in the previous section. It is plausible that these shock waves superimposed on the existing planar capillary waves, propagating at 24 kHz along the interphase boundary resulting in nonlinear wave interactions, thus distorting their propagation frequency as seen in Fig. 2c (refer to Supplementary video 2). With the increase in acoustic cycles, the wavelength further increased to approx. 150 µm, pushing the droplet to a critical/unstable state at *t* = 1.29 ms (Fig. 3b) coinciding with the 1st subharmonic, *f_0_*/2, culminating in atomization and ejection from the horn tip at *t* = 1.46 ms. The medium angle (MA) droplet exhibited a similar evolution pattern but over a slightly longer timescale. At *t* = 1.33 ms, faint ripples started to appear with *λ_CW_* close to 90 µm, indicating the onset of capillary waves travelling close to the driving frequency of 24 kHz. By *t* = 1.55 ms, the waves displayed an increase in *λ_CW_* by almost 40 µm reaching up to 130 µm. This was then followed by a swift transition to instability ≈ *f_0_/2* (*t* = 1.60 ms) and subsequent atomization observed at *t* = 1.75 ms. The high angle (HA) droplet demonstrated the most delayed response. At *t* = 1.61 ms, the ripples appeared at the interphase boundary with a wavelength of approx. 92 µm, which further increased with surface oscillations by 33 µm at *t* = 1.87 ms. The droplet then entered an unstable phase at *t* = 1.98 ms, with atomization initiating at *t* = 2.12 ms.

For LA, the wavelength increased sharply with the number of acoustic cycles as shown in the corresponding wavelength plot exhibiting a slope of ∼88°, reflecting the rapid onset of instability possibly due to the chaotic pattern caused by interference of axisymmetric and shock waves as discussed in the previous section. MA and HA, however, showed a more gradual increase, with a slope of ∼84°, consistent with the observed stability in the corresponding frames in the image sequence. This occurred because a smaller mass of the LA droplet enabled more frequent cavitation events near the sonotrode surface at a specific driving amplitude, resulting in a faster interaction between planar and spherical waves. In contrast, the larger liquid volumes in MA and HA cases allowed shock waves to travel farther, reducing the ratio of cavitation events to liquid volume. Importantly, in all cases, atomization occurred when the wavelength of capillary waves changed to subharmonic regime followed by the emergence of chaotic patterns at the liquid–air interface, thus coinciding with the emission of subharmonic signatures by periodic shock waves.

#### 3.1.2 Isopropyl alcohol

Fig. 3c captured the dynamic response of an IPA droplet (also at 20 % input power), illustrating some variations in behaviour compared to DIW due to its different physical properties, specifically larger viscosity and vapour pressure (see Table 1). At the outset *t* = 0 ms, the droplet was at rest. As ultrasonic vibrations were applied (*t* = 1.21 ms), the LA droplet began to oscillate at the applied excitation frequency, leading to the emergence of capillary waves. These waves, with *λ_CW_* approx. 45 µm, were smaller than those observed in DIW. The phase velocity of these capillary waves was also observed to be lower, around 1.4 m/s. Once again, the initial propagation frequency of these ripples coincided with the driving frequency from the acoustic source. The wavelength of the capillary waves then grew to 68 ± 4 µm at *t* = 1.43 ms with a slope of ∼88°, possibly due to the decrease in the microbubble oscillation frequency below the excitation frequency of 24 kHz, as seen in the case of DIW. The wavelength further increased, pushing the droplet to an unstable state at *t* = 1.49 ms leading to atomization at *t* = 1.58 ms. The MA droplet, on the other hand, began to show faint ripples at *t* = 1.43 ms, with a wavelength of approx. 59 µm. This quick onset of surface waves progressed to an increase in *λ_CW_* by almost 26 µm at *t* = 1.52 with a slope close to 84°, similar to the LA case, followed by a transition to instability (*t* = 1.61 ms) and atomization at *t* = 1.67 ms. The HA droplet showed the most delayed response among the three, with ripples forming at *t* = 1.42 ms and *λ_CW_* of about 71 µm. The *λ_CW_* then increased by almost 30 µm at *t* = 1.55 ms with a slope of 83°, entering an unstable phase (*t* = 1.67 ms) and initiating atomization at *t* = 1.75 ms. Thus, the mechanism of capillary wave formation and increase in wavelength followed by transition to instability and subsequent atomization in IPA remained fairly similar to those in DIW, although more chaotic, for LA droplets. A similar relationship between the wavelength and frequency of propagating capillary waves has been reported previously in several studies using Kelvin's equation: $\lambda_{\mathrm{CW}}={\left(2\pi \sigma /\rho {f_{\mathrm{CW}}}^{2}\right)}^{1/3}$[35], [36], [37], [38], [39], [40], where *λ*_CW_ is the capillary wavelength, *σ* represents the liquid's surface tension, *ρ* is the density, and *f*_CW_ is the frequency of the capillary waves. Interestingly, this theoretical framework aligns with our observations of surface wave characteristics evolving from stable harmonic to unstable subharmonic frequencies for droplets of both DIW and IPA as seen in Table 2. The differences observed between DIW and IPA can be attributed to their distinct physical properties that directly affect the capillary wavelength as per Kelvin's equation. It is important to note here that the frequency of the produced surface waves can vary depending on the impinging angle of ultrasonic wave with the liquid free surface as explained by Mahravan et al. [41].

**Table 2:** Frequency of ultrasonically-induced surface waves in DIW and IPA measured experimentally and theoretically during stable and unstable regime.

**Liquids**	**Travelling surface waves**							**Frequency (*f*_CW_)**											
	**Velocity** ***V*_CW_ (m/s)**	**Wavelength** ***λ*_CW_** **(µm)**						**Experimental (kHz)**						**Theoretical from Kelvin’s Eq. (kHz)**					
		**Stable**			**Unstable**			**Stable**			**Unstable**			**Stable**			**Unstable**		
		LA	MA	HA	LA	MA	HA	LA	MA	HA	LA	MA	HA	LA	MA	HA	LA	MA	HA
**DIW**	2.1 ± 0.1	85 ± 5.4	90 ± 4.6	92 ± 5.8	151 ± 5.8	156 ± 5.8	176 ± 5.3	23.6 ± 1.2	23.3 ± 0.9	23.9 ± 1.2	13.2 ± 0.4	13.4 ± 0.4	12.5 ± 0.3	28.5 ± 2.2	26.1 ± 1.6	25.2 ± 2.2	11.4 ± 0.5	10.9 ± 0.5	9.1 ± 0.3
**IPA**	1.4 ± 0.3	44 ± 4.2	59 ± 3.6	71 ± 4.5	91 ± 3.6	110 ± 5.1	133 ± 4	25.1 ± 2	23.7 ± 1.2	24 ± 1.2	12.1 ± 0.4	12.7 ± 0.5	12.8 ± 0.3	45.9 ± 5.3	29.3 ± 2.2	22.2 ± 1.7	15.3 ± 0.7	11.5 ± 0.6	8.6 ± 0.3

Some prior studies have reported that the capillary waves initially vibrate with a frequency equal to the forcing frequency, before reaching the resonance (subharmonic) frequency [42], [43]. Table 2 clearly shows that for HA droplets (with close to normal wave incidence), the experimental frequency of the observed stable capillary wave is initially in good agreement with the theoretical frequency derived from Kelvin’s equation. However, for LA and MA droplets i.e. when the incident angle changes, the calculated theoretical capillary wave frequency starts to diverge from the experimental observations for both stable and unstable regimes. This is because at non-normal incidence, the impinging ultrasound interacts with the liquid surface in a more complex manner, generating non-axisymmetric wave modes due to increased cavitation activity emitting shock waves from subharmonic bubble collapses [20], [44]. The observed shift from the driving frequency ($f_{o}$) to subharmonic mode ($f_{o}$/2) prior to liquid breakup and ejection of droplets is consistent with the findings from the Mathieu equation described by Banjamin and Ursell [12]. The frequency transition is primarily caused by bubbles implosions generating non-axisymmetric azimuthal wave modes on the liquid surface rather than parametric resonance from the source. These dynamics lead to unstable and chaotic behaviour of the free surface, as previously reported by James et al. [45] and earlier in Section 3.1.

It is interesting to note the differences in the timescales (Δ*t*) of surface wave evolution between the DIW and IPA droplets, which is markedly influenced by their surface tension, vapour pressure and viscosity. Our previous studies demonstrated the importance of these physical parameters on cavitation activity and bubble dynamics in similar liquids [24], [25]. DIW exhibited a more gradual transition from stability to atomization, spanning from *t* = 1.61 ms to 2.12 ms (Δ*t* ∼ 500 µs) for HA droplets. Conversely, IPA demonstrated accelerated destabilization, compressing the transition timeframe between 1.42 ms to 1.75 ms (Δ*t* ∼ 300 µs). This rapid transition in IPA likely stemmed from its reduced ability to dampen ultrasonic energy (lower surface tension), resulting in quicker surface destabilization. On the other hand, the delayed response in HA droplets in comparison to MA and LA counterparts, observable in both liquids, suggested that initial droplet size and cavitation activity within affected the dynamics of wave evolution. Corroborating this observation, recent X-ray synchrotron studies [9], [10] confirmed the presence of subharmonic capillary waves immediately preceding atomization. These observations elucidated that subharmonic capillary waves represented the most unstable state, likely induced by the interference of stable (planar) capillary waves and omnidirectional shock waves emitted from subharmonic microbubble oscillation and collapses occurring near the liquid air interface as observed in Fig. 2d. This transition plays a pivotal role in the atomization process. Thus, we hypothesize that the observed changes in capillary wave characteristics might result from the subharmonic bubble implosions producing shock waves, which most likely lead to the introduction of nonlinearities into the system.

#### 3.1.3 Glycerol

The evolution of surface waves in glycerol (Gly) was, however, drastically different. At a low input power setting of 20 %, there were no visible ripples, and atomization did not occur. Hence, the experiment was performed at 100 % power (peak-to-peak amplitude of 125 µm) instead, as shown in Fig. 3d. Unlike DIW and IPA, where capillary waves dominated the initial stages, the high viscosity of glycerol led to a unique sequence. For all droplet shapes (LA, MA, HA), surface disturbances on the droplet surface marked the initiation beyond *t* = 5 ms. However, these disturbances did not develop into classical waves. Instead ‘viscous fingers’ emerged in the case of LA and MA droplets, as described elsewhere [46], [47]. These ‘fingers’ then elongated into thin filaments, ejected with velocities up to 12 m/s, eventually rupturing into smaller fragments, a behaviour previously linked to cavitation activity under the liquid dome, as seen in [9]. Thicker droplets (HA) exhibited a similar sequence but with a delayed evolution.

The results from this section demonstrated the importance of the subharmonic surface waves in the atomization process. However, the subsequent evolution and breakup of the liquid droplets might involve additional mechanisms beyond the initial shock wave and interphase boundary interaction. For instance, the growth and rupture of viscous ‘fingers’ observed in the case of glycerol suggest that interfacial tension and shearing forces may play a more dominant role in the atomization process for highly viscous liquids. Furthermore, the transition from the fundamental frequency to the subharmonic regime, as observed in DIW and IPA, implied that the shock waves may be responsible for introducing nonlinearities into the system, altering the characteristics of the capillary waves and facilitating droplet destabilization and breakup. However, this nonlinear behaviour, driven by the interaction between shock waves and the liquid surface warrants further investigation to fully understand the complexities of the atomization process driven by cavitation. Thus, in the next section, we characterize *in-situ* the acoustic emissions upon atomization within the droplets of different liquids to decipher the role of shock waves in producing droplet mist.

### 3.2 Acoustic emissions

**Fig. 4:**
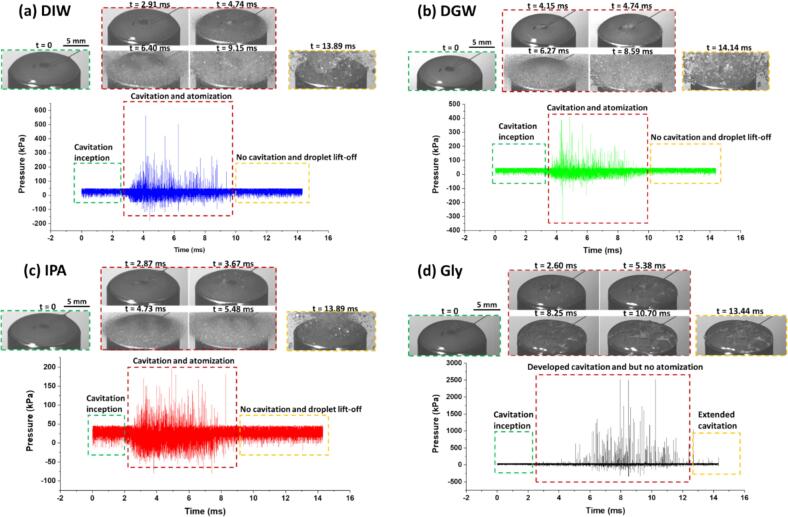
*In-situ* detection of acoustic emissions showing pressure–time profile with captured cavitation and/or atomization events in (a) deionized water, (b) degassed water, (c) isopropyl alcohol and (d) glycerol, under ultrasonic excitation at 20% input power. Note the difference in the pressure (Y) axis scale.

Fig. 4a shows the synchronized high-speed images and recorded acoustic emissions, providing a distinctive insight into the intricacies of ultrasonic atomization of a DIW droplet on a 14 mm horn tip (refer to supplementary video 6). The first captured frame shows the droplet (*t* = 0 ms) with pre-existing bubbles and the baseline pressure in the time-domain. However, around *t* ≈ 1.94 ms, the high-speed camera captured the formation of cavitation bubbles and microbubble clusters with the liquid drop, while the pressure time plot exhibited rising pressure signatures, indicating the growth and collapse of these bubbles. Note that these cavitating bubbles are likely the source of the high-energy shock waves. Although not directly visualized in this study, these shock waves had been resolved and investigated in our previous studies [23], [24]. Sharp peaks captured in the time domain after every collapse indicated the propagation of these shock waves through the liquid medium. However, it is important to note that there could be instances when a shock pressure peak was absent in the time domain affecting the periodicity of shock wave fronts released upon bubble cloud collapse, which has been referred to as non-collapse deflation elsewhere [22]. As cavitation intensified (*t* ≈ 1.94 ms to 4.74 ms), the corresponding images show the formation of tiny secondary droplets from the edges of horn-liquid interface with shock pressure peaks reaching up to 500 kPa contributing to the initial stages of atomization. Between *t* ≈ 4.74 ms and *t* ≈ 9.15 ms, a decrease in pressure magnitude was observed possibly signifying a decline in cavitation activity as most of the droplet had already been atomized and lifted off (*t* ≈ 9.15 ms onwards). To eliminate the effects of dissolved gases (present within the DIW droplet) that could have an influence on atomization characteristics, we conducted experiments with degassed water (DGW) as shown in Fig. 4b. The high-speed image sequence and pressure–time plot showed almost the same features in terms of spatial and temporal response of ultrasound on atomization (refer to supplementary video 7). The only notable difference observed was a smaller number of concentrated cavitation bubbles formed within the degassed liquid unlike DIW where the microbubbles were distributed across the whole of horn surface.

Fig. 4c illustrates the atomization sequence and acoustic emissions for IPA (refer to supplementary video 8). At *t* = 0 ms, the pressure–time plot shows a baseline pressure with droplet at rest on the horn tip. As the ultrasonic excitation began, the pressure started to fluctuate, marking the onset of cavitation. Beyond *t* ≈ 2 ms, the pressure peaks increased with the initial formation of microbubbles and capillary waves. At *t* = 3.67 ms, these pressure peaks became more pronounced, reaching up to 170 kPa, as the microbubbles coalesced and imploded. The sequence further intensified at *t* ≈ 5 ms, with shock pressure peaks surging to around 200 kPa, signalling the violent collapse of cavitation clouds and the consequent generation of atomized droplets. Both cavitation and atomization occurred until *t* ≈ 9 ms, with subsequent decrease in pressure spikes returning to near-baseline levels, suggesting the cessation of both cavitation activity and atomization characterized by droplet lift-off as seen in case of DIW and DGW.

Glycerol, as depicted in Fig. 4d (refer to supplementary video 9), presents a completely different behaviour compared to DIW and IPA as expected. Despite ongoing cavitation in glycerol (pressure peaks persisting beyond *t* ≈ 5 ms), the image throughout this timeframe showed no signs of atomization (except a single ligament formation and droplet pinch-off observed at *t* ≈ 8 ms). This suggests that the high viscosity (around 950 mPa.s compared to 1 mPa.s for water and 2.4 mPa.s for isopropyl alcohol) dampened the shock waves from collapsing bubbles, which were crucial for breaking up droplets in less viscous liquids. It appeared that the forces exerted on the glycerol droplet during bubble collapse were insufficient to overcome its viscous forces and initiate breakup (atomization threshold). Furthermore, the pressure–time plot hinted at potentially longer-lasting cavitation in glycerol (pressure peaks continued beyond the timeframe typically observed for atomization in DIW and IPA), likely due to the slow diffusion of dissolved air/gases. Note, for comparison purposes, the pressure–time plots for all liquids were depicted until ∼14 ms. However, in case of glycerol (Fig. 4d), cavitation activity extended much beyond 14 ms without any visual signs of atomization. In essence, cavitation in glycerol was rendered ineffective by the double action of viscosity: dampening shock waves and prolonging bubble existence as seen in [24]. Therefore, glycerol took significantly longer to start atomizing in the form of ejected thin filaments (as shown in supplementary video 12 in [9]) than the limited time frame depicted in Fig. 4d. Thus, the pressure–time behaviour during this event may not fully capture the complete picture for such longer durations due to acoustic monitoring limitations in this study.

**Fig. 5:**
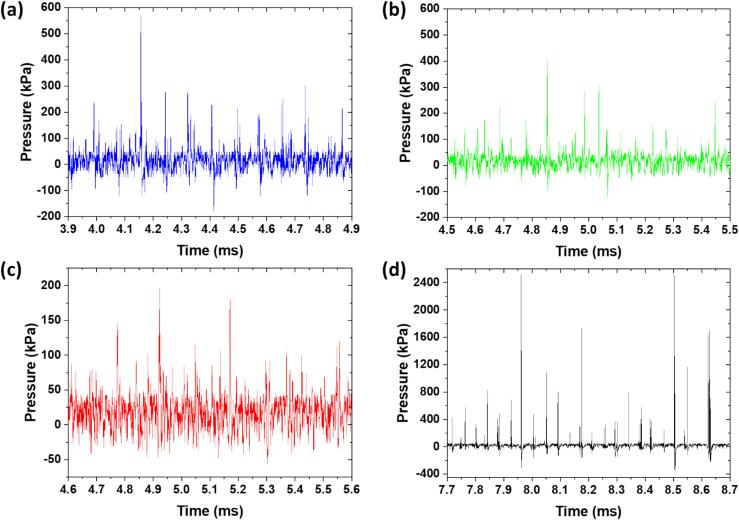
Pressure vs time plots of (a) DIW, (b) DGW, (c) IPA and (d) Gly showing periodicity of shock spikes.

Among the four studied liquids, IPA exhibited the earliest signs of both cavitation and atomization, followed by deionized water DIW and DGW, and then glycerol, where no atomization was observed. Notably, for glycerol, the appearance of the shock pressure peaks in the pressure–time domain plot occurred after almost twice the time compared to the other three liquids. Moreover, the registered pressure is almost 5 times more than for the other liquids, which is possibly due to multiple shock fronts accumulating in small liquid droplet volume followed by rapid attenuation of the shock wave intensity in the extended cavitation regime (Fig. 4d). This is likely due to the large population of cavitation bubble clouds that hinders the effective propagation of shock fronts and attenuates their strength due to distinct physical properties as discussed elsewhere [24], [25]. Shielding in other liquids is comparatively low resulting in breakup and atomization. We suggest that the appearance of subharmonic capillary waves in case of DIW and IPA (Fig. 3b and 3c) might be linked to the periodicity of shock pressure peaks generated from subharmonic bubble collapse. Fig. 5 shows the pressure–time plots for the four liquids indicative of major and minor peaks. While minor peaks were mainly associated with the fundamental frequency, the equidistant major peaks contributed to the subharmonics [24]. It can be seen that for DIW, DGW and Gly, shock (major) spikes appeared every 80 µs (∼2 acoustic cycles), while for IPA, the peaks were separated by 2 to 3 acoustic cycles. Due to high vapour pressure and lower surface tension of liquids like IPA and ethanol, they generate large cavitation cloud structures with pressure magnitudes approximately half of those observed in water within the cavitation zone as noted in [24] leading to faster atomization.

While cavitation occurred in all four scenarios, the ability of the liquid to propagate the shock waves and the forces exerted on the droplet surface during bubble collapse were critically affected by the viscosity in the case of glycerol and the surface tension and vapour pressure in IPA. Moreover, it can be observed in supplementary videos 6 to 8 that atomization persisted in water and IPA as long as cavitation activity was present, and the droplet lift-off from the horn tip coincided with a reduction in cavitation intensity. This suggests a causal link between cavitation-induced shock waves and the atomization process. It appears that the shock waves play a role in the atomization of large liquid droplets, providing the necessary energy to disperse the liquid into fine aerosols.

### 3.3 Spectral response during atomization

**Fig. 6:**
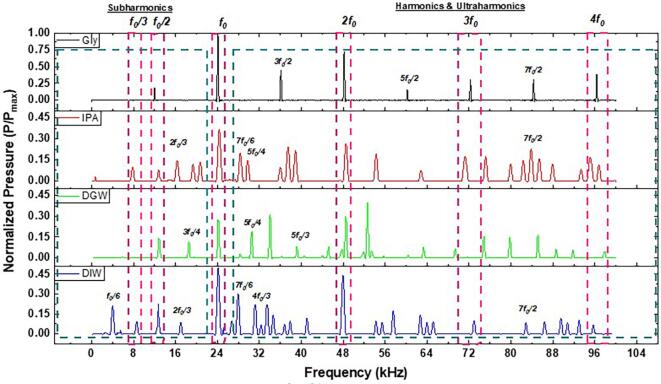
Acoustic pressure (normalized) – frequency spectrum in the liquids studied during ultrasonic atomization obtained from Fourier transformed pressure–time data sampled at 20 MHz.

In order to have a better understanding of acoustic emissions triggering the formation of surface waves (axisymmetric planar as well as non-axisymmetric subharmonic azimuthal waves), where their interference leads to droplet breakup and eventual atomization, we now turn our attention to the Fourier transformed pressure-frequency spectrum (normalized to maximum pressure, *P*_max_) of the four liquids captured *in-situ*. Fig. 6 shows the spectral characterization of the cavitation activity observed during atomization. It should be emphasized here that the variation in amplitude of the acoustic signals shown in Fig. 5 should result in the emergence of broadband noise in the FFT spectrum as recently highlighted by Yasui [48]. Since the acoustic measurement in this study was performed in close proximity to the horn tip, where the cavitation/supercavitation cloud forms under ultrasonic excitation [49], the raw acoustic spectrum was found to be quite chaotic (see Fig. S2 in the supplementary material). Especially for DIW, DGW and IPA droplets, the spectrum was too noisy making it difficult to discern the dominant frequencies of interest primarily associated with periodic shock pressure peaks, cavitation bubble collapse and bubble–bubble interactions. Therefore, it was necessary to post-process the deconvoluted data by subtracting the noise floor and smoothening the spectrum. The spectrum showed the presence of multiple subharmonics, harmonic and ultra-harmonic peaks in the studied liquids. Harmonics (2*f_0_*, 3*f_0_*, and 4*f_0_*) and ultra-harmonic (3*f_0_*/2, 5*f_0_*/2 and 7*f_0_*/2) and subharmonic (*f_0_*/2) peaks were observed for all cases. DIW, DGW and IPA displayed additional subharmonic and ultra-harmonic frequencies (3*f_0_*/4, 2*f_0_*/3, *f_0_*/3 and *f_0_*/6, 7*f_0_*/6, 5*f_0_*/4, 5*f_0_*/3 and 4*f_0_*/3) as opposed to glycerol. These frequencies were related to the less viscous nature of these fluids, which allowed for more complex bubble dynamics and unconstrained vigorous individual microbubble and bubbly cluster oscillations [25], [50]. These varied oscillating modes permitted energy transfer across a broad spectrum of frequencies with attenuated pressure magnitudes, impacting droplet breakup and promoting atomization. Glycerol, notably, exhibited sharper and more intense fundamental and harmonic peaks than IPA, DIW and DGW. This can be tied to its higher viscosity. In glycerol, the viscous forces dampen the cavitation bubble oscillations more effectively than in less viscous liquids, leading to lesser energy dissipation into the medium due to the cavitation shielding effect as a result of populated cavitation structures in confined liquid volume and thus resulting in more pronounced spectral peaks at harmonic frequencies [24]. Although, the high viscosity of glycerol promoted the sustained oscillation of microbubble clusters, the damping characteristics also inhibited the formation of any wave patterns on the liquid surface unlike water and IPA.

The roles of harmonics, ultraharmonics and subharmonics emissions in atomization are essential. These emissions indicate the presence of periodic shock waves as reported elsewhere [21]. Ultraharmonics are a signature of the higher-order microbubble cluster oscillations that could also result in harmonics from the subharmonic frequencies generating periodic shock fronts [51] that causes capillary wave formation on the liquid surface [52]. Subharmonics, on the other hand, are typically associated with the nonlinear cavitation and periodic shock wave emissions [21], [22], [24], [53], [54] resulting from the violent collapses of microbubble clusters and bubble cloud that generate intense local pressure spikes facilitating the rapid breakup of the liquid into fine droplets as discussed in Section 3.1. These subharmonic peaks in the spectrum were a direct measure of the energy released during these implosive events. Furthermore, the appearance of these subharmonics, particularly at lower fractions such as *f_0_*/2, 2*f_0_*/3, *f_0_*/3, and *f_0_*/6, suggested that the liquid was exhibiting resonant behaviour only at specific modes that were characteristic of Faraday waves [8], [9], [10], [55]. This resonant response was closely tied to the Faraday instability, where the periodic modulation of the liquid surface by the external vibration led to the preferential amplification of certain wave modes, resulting in the observed subharmonic frequencies of the azimuthal waves. Our observations of subharmonic frequency during ultrasonic atomization bears some comparison with the findings of Wang et al. [56], albeit involving a different experimental approach and frequency regimes. In their paper, it was observed that under high-frequency (1–3 MHz) ultrasonic atomization, the formed beads corresponded to half of the ultrasound wavelength and the dominant oscillation frequencies were subharmonic to the driving. Our study, conducted at much lower frequencies (∼24 kHz) also showed the presence of subharmonic frequencies of the unstable surface waves prior to droplet formation, but through a different mechanism. Wang et al. [56] demonstrated that the subharmonic frequencies were linked to the natural resonance frequencies of the beads as predicted by Rayleigh's equation [57] for liquid drop oscillations. In contrast, our synchronized high-speed visualization and acoustic emission results revealed that these subharmonic frequencies emerge primarily from the interference between non-axisymmetric omnidirectional shock waves and the initially stable harmonic capillary waves. This is particularly evident in our experimental data (Fig. 3 and Fig. 6), where the surface waves initially propagate at the driving frequency (24 kHz), consistent with Kelvin's equation predictions (Table 2), before transitioning to subharmonic modes (∼12 kHz) due to introduction of nonlinearities in the system by oscillating and imploding cavitation bubbles. While Wang et al. [56] reported non-periodic/quasiperiodic droplet bursting events near the peaks of bead oscillations, our study demonstrated that the transition from harmonic to subharmonic frequencies follows a systematic pattern driven by cavitation activity. These contrasting observations suggest that while subharmonic frequencies are a common feature in ultrasonic atomization across a wide range of frequencies, the underlying mechanisms can vary significantly depending on the dominant physical phenomena at play instigated by the nonlinear interaction between liquid free surface and acoustic waves. The relationship between the observed subharmonics and surface wave instability can be further elucidated by considering the formation and excitation of axisymmetric (planar) waves, which are linked to harmonic frequencies in the acoustic spectrum. The frequency perturbation then occurs as a result of cavitation activity though nonlinear interactions of the subharmonic components. In addition to the physical properties of the liquid, such as viscosity, surface tension, and density that was already discussed elsewhere [8], liquid/film volume could also play a crucial role in determining its response to external perturbations, which could alter the propagation of surface waves, thereby affecting the spectral characteristics observed in the frequency spectrum.

### 3.4 Role of shock waves

**Fig. 7:**
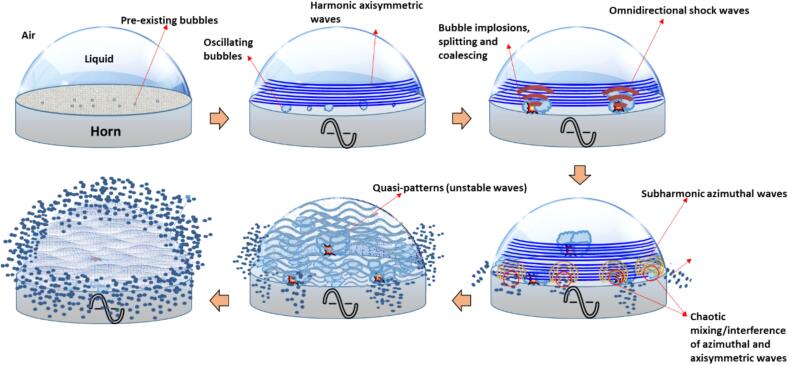
Schematic depiction of the triggering mechanism of atomization of a sessile liquid droplet under ultrasonic excitation.

Following detailed observations of capillary wave characteristics, cavitation dynamics and detection of shock wave emissions within a liquid droplet under ultrasonic excitation, we now delineate the underlying triggering mechanism with shock waves playing a dominant role in the atomization process through a schematic description as shown in Fig. 7. The atomization mechanism of a liquid droplet on ultrasonic horn proceeds through several distinct stages. Initially, pre-existing microscopic bubbles are distributed throughout the liquid droplet resting on the horn surface. It can be suggested that in the absence of these pre-existing bubbles, there will be a need to form cavitation nuclei, which may delay the onset of cavitation. When ultrasonic excitation begins, two simultaneous phenomena occur: the droplet surface develops harmonic axisymmetric waves (visible as planar circumferential patterns in Fig. 2b) similar to those observed by Panda et al. [15], Noblin et al. [58] and Vukasinovic et al. [59], while if pre-existing bubbles are present, they begin to oscillate in response to the acoustic field. As the process continues, these oscillating bubbles undergo complex dynamics including implosion, splitting, and coalescence events. These violent bubble dynamics then generate omnidirectional shock waves that propagate through the liquid and manifest as spherical wave patterns upon reaching the liquid–air interface (see Fig. 2b). These spherical wave patterns exhibit larger wavelengths (formed by subharmonic bubble collapses) compared to the axisymmetric waves (induced by the ultrasonic source) showing the emergence of subharmonic azimuthal waves, which were previously observed [15], [58], [59] but whose origin remained unclear until now. The interaction between the initial harmonic axisymmetric waves and these shock wave-induced spherical patterns creates regions of constructive and destructive interference, resulting in quasi-patterns characterized by unstable wave structures similar to those observed by Edwards and Fauve [60]. At this stage, the wave dynamics transition from being purely harmonic to include subharmonic components, particularly visible in the formation of subharmonic azimuthal waves near the liquid-horn-air interface. The final stage of the process occurs when these interfering wave patterns create regions of intense chaotic mixing, where the superimposition of axisymmetric and azimuthal waves leads to local instabilities [15]. These instabilities ultimately trigger droplet atomization, primarily initiating at the liquid-horn-air interface where the wave interference is most pronounced.

It is worth noting here that the transition from stable planar waves to unstable chaotic patterns, accompanied by a frequency shift from driving to subharmonic frequencies, bears similarity to classical Faraday wave instability [61]. However, our findings suggest that shock waves are the key mediator of this interaction offering a new understanding of how azimuthal subharmonic modes emerge, thus providing the energy necessary for the formation of chaotic patterns and subsequent droplet atomization.

## 4 Conclusions

In this work, we have provided direct experimental validation that cavitation-induced shock waves play a pivotal role in initiating surface wave instability and subsequent atomization of liquids under ultrasonic excitation. *In-situ* acoustic emission detection has confirmed that the atomization of liquid droplets is sustained by ongoing cavitation activity, with the intensity of cavitation directly influencing the process. The presence of subharmonic frequency peaks in the acoustic emission spectra reflects the transition from stable axisymmetric planar (fundamentally driven) waves to unstable capillary (sub-harmonically driven) waves formed on the interphase boundary upon superimposition with the omnidirectional cavitation-induced shock waves. The viscosity of the liquid critically influences its ability to propagate these shock waves, where the efficient propagation in low-viscosity liquids like water and isopropyl alcohol leads to rapid surface destabilization and atomization, while the high viscosity of glycerol dampens the shock waves, inhibiting wave instability. The transition from fundamental to subharmonic frequencies suggests that periodicity of shock wave-induced nonlinearities alter the capillary wave characteristics, facilitating breakup. While shock waves could initiate atomization in certain liquids, droplet breakup in highly viscous liquids may involve additional mechanisms associated with viscous fingering and shearing of ligaments.

## CRediT authorship contribution statement

**Abhinav Priyadarshi:** Writing – review & editing, Writing – original draft, Visualization, Validation, Software, Methodology, Investigation, Formal analysis, Data curation, Conceptualization. **Paul Prentice:** Writing – review & editing, Supervision, Software, Resources, Methodology, Investigation. **Dmitry Eskin:** Writing – review & editing, Supervision, Resources, Funding acquisition. **Peter D. Lee:** Writing – review & editing, Supervision, Funding acquisition. **Iakovos Tzanakis:** Writing – review & editing, Supervision, Resources, Funding acquisition.

## Declaration of competing interest

The authors declare that they have no known competing financial interests or personal relationships that could have appeared to influence the work reported in this paper.
