# Supplementary material for: Synchronized acoustic emission and high-speed imaging of cavitation-induced atomization: The role of shock waves
Source: Ultrason Sonochem. 2025 Jan 18;113:107233. doi: 10.1016/j.ultsonch.2025.107233 (PMC11795154; doi:10.1016/j.ultsonch.2025.107233)
Supplement: Supplementary Data 10 [file mmc10.docx]

**Synchronized acoustic emission and high-speed imaging of cavitation-induced atomization: The role of shock waves**

Abhinav Priyadarshi1*, Paul Prentice^2^, Dmitry Eskin^3^, Peter D. Lee^4,5^, Iakovos Tzanakis1,6

1School of Engineering Computing and Mathematics, Oxford Brookes University. Oxford, UK

^2^Cavitation Laboratory, Centre for Medical and Industrial Ultrasonics, University of Glasgow, Glasgow, UK

3Brunel Centre for Advance Solidification Technology (BCAST), Brunel University of London, Uxbridge, UK

^4^Department of Mechanical Engineering, University College London, London, UK

^5^Research Complex at Harwell, Harwell Campus, Oxfordshire, UK

^6^Department of Materials, University of Oxford, Oxford, UK

**Supplementary Figures**

**Fig. S1** Sensitivity of FOH calibrated between 1-30 MHz bandwidth frequencies.

**Fig. S2** Raw/unprocessed pressure-frequency spectrum in the studied liquids showing the broadband noise and chaotic nature of the signal.
